# Late

**Published:** 1873-04

**Authors:** 


### The Bistoury.

ELMIRA, N. Y., APRIL, 1873.

The Bistoury is published Quarterly, upon the 1st of April, July, October and January, at Fifty Cents a year, in advance.

### LATE.

By reason of the disappointment of the printers of this journal, in not receiving the press upon which this new edition of the Bistoury is printed, at the time contracted for, our journal is delayed far beyond the usual time for its publication. We hope our readers will excuse this delay, inasmuch as it could not be prevented, and that they will rejoice with us in our improved appearance, by reason of new type, paper and press.

